# Supplementary material for: Cell-free RNA Signatures Derived from the Tumor Microenvironment Predict Outcomes of CAR-T Therapy in Large B Cell Lymphoma
Source: medRxiv. 2026 Mar 18:2026.03.16.26348550. Preprint. [Version 1] doi: 10.64898/2026.03.16.26348550 (PMC13015665; doi:10.64898/2026.03.16.26348550)
Supplement: 1 [file NIHPP2026.03.16.26348550V1-supplement-1.pdf]

medRxiv preprint doi: https://doi.org/10.64898/2026.03.16.26348550; this version posted March 18, 2026. The copyright holder has placed this preprint (which was not certified by peer review) in the Public Domain. It is no longer restricted by copyright. Anyone can legally share, reuse, remix, or adapt this material for any purpose without crediting the original authors.

Supplementary Materials For

Cell-free RNA Signatures Derived from the Tumor Microenvironment Predict Outcomes of CAR-T Therapy in Large B Cell Lymphoma

Authors: Conor Loy[1,*], Glory Agun[1,*], Katie Maurer[2,3], Anna Bosch Vilaseca[2], Daria Potapova[2], Caron A. Jacobson[2,3], Jerome Ritz[2,3], and Iwijn De Vlaminck[1,#]

Affiliations:
[1] Nancy E. and Peter C. Meinig School of Biomedical Engineering, Cornell University; Ithaca, 14850, USA.
[2] Department of Medical Oncology, Dana-Farber Cancer Institute; Boston, 02215, USA
[3] Department of Medicine, Harvard Medical School; Boston, 02115, USA
* These authors contributed equally
[#] Correspondence: vlaminck@cornell.edu

medRxiv preprint doi: https://doi.org/10.64898/2026.03.16.26348550; this version posted March 18, 2026. The copyright holder has placed this preprint (which was not certified by peer review) in the Public Domain. It is no longer restricted by copyright. Anyone can legally share, reuse, remix, or adapt this material for any purpose without crediting the original authors.

Table S1. Patient demographics.

	Total n = 91 (%)	Cohort 1 n = 28 (31)	Cohort 2 n = 28 (31)	Cohort 3 n = 35 (38)	*p-value*
**Age at infusion**					
Median (range)	65 (24 - 83)	66 (33 - 83)	64 (39 - 76)	65 (24 - 77)	*0.77^*
≤65	49 (54)	13 (46)	16 (57)	20 (57)	*0.67‡*
>65	42 (46)	15 (54)	12 (43)	15 (43)	
**Diagnosis**					
De novo DLBCL	67 (74)	11 (39)	24 (86)	32 (91)	*< 0.001‡*
FL	4 (4)	3 (11)	1 (4)	-	
HGBL	2 (2)	2 (7)	-	-	
PMBCL	1 (1)	-	-	1 (3)	
tCLL	1 (1)	1 (4)	-	-	
tFL	14 (15)	9 (32)	3 (11)	2 (6)	
tMZL	2 (2)	2 (7)	-	-	
**Subtype**					
Germinal Center	42 (46)	16 (57)	13 (46)	13 (37)	*0.41‡*
Non-GCB	32 (35)	8 (29)	10 (36)	14 (40)	
Missing	*17 (19)*	*4 (14)*	*5 (18)*	*8 (23)*	
**Stage**					
I	5 (5)	-	1 (4)	4 (11)	*0.0049‡*
II	5 (5)	-	1 (4)	4 (11)	
III	16 (18)	4 (14)	10 (36)	2 (6)	
IV	62 (68)	24 (86)	16 (57)	22 (63)	
Missing	*3 (3)*	*-*	*-*	*3 (9)*	
**CNS lymphoma**					
No	47 (52)	11 (39)	11 (39)	25 (71)	*0.88‡*
Yes	9 (10)	1 (4)	2 (7)	6 (17)	
Missing	*35 (38)*	*16 (57)*	*15 (54)*	*4 (11)*	
**Double-hit**					
No	76 (84)	20 (71)	24 (86)	32 (91)	*0.038‡*
Yes	12 (13)	7 (25)	4 (14)	1 (3)	
Missing	*3 (3)*	*1 (4)*	*-*	*2 (6)*	
**Triple-hit**					
No	85 (93)	25 (89)	27 (96)	33 (94)	*0.20‡*
Yes	3 (3)	2 (7)	1 (4)	-	
Missing	*3 (3)*	*1 (4)*	*-*	*2 (6)*	
**ECOG at diagnosis**					
0	24 (26)	6 (21)	7 (25)	11 (31)	*0.52‡*
1	41 (45)	15 (54)	14 (50)	12 (34)	
2	8 (9)	1 (4)	3 (11)	4 (11)	
Missing	*18 (20)*	*6 (21)*	*4 (14)*	*8 (23)*	
**IPI**					
0	1 (1)	-	1 (4)	-	*0.034‡*

medRxiv preprint doi: https://doi.org/10.64898/2026.03.16.26348550; this version posted March 18, 2026. The copyright holder has placed this preprint (which was not certified by peer review) in the Public Domain. It is no longer restricted by copyright. Anyone can legally share, reuse, remix, or adapt this material for any purpose without crediting the original authors.

Table S1. Patient demographics.

		Cohort			
	**Total** n = 91 (%)	**1** n = 28 (31)	**2** n = 28 (31)	**3** n = 35 (38)	***p-value***
1	10 (11)	1 (4)	3 (11)	6 (17)	
2	15 (16)	2 (7)	6 (21)	7 (20)	
3	26 (29)	15 (54)	7 (25)	4 (11)	
4	23 (25)	5 (18)	10 (36)	8 (23)	
5	4 (4)	1 (4)	1 (4)	2 (6)	
Missing	*12 (13)*	*4 (14)*	*-*	*8 (23)*	
**Prior SCT**					
Allo	1 (1)	1 (4)	-	-	*0.17*‡
Auto	22 (24)	7 (25)	10 (36)	5 (14)	
Neither	66 (73)	20 (71)	18 (64)	28 (80)	
Missing	*2 (2)*	*-*	*-*	*2 (6)*	
**Prior chemotherapy**					
No	9 (10)	4 (14)	-	5 (14)	*0.11*‡
Yes	82 (90)	24 (86)	28 (100)	30 (86)	
**No. of pre-infusion therapies**					
Median (range)	3 (1 - 10)	3 (1 - 10)	3 (1 - 5)	2 (1 - 5)	*0.026*‡
Missing	*7 (8)*	*2 (7)*	*-*	*5 (14)*	
**Best Response Rate**					
**CR**	75 (82)	21 (75)	24 (86)	30 (86)	
**PR**	6 (7)	2 (7)	1 (4)	3 (9)	
**SD/PD**	10 (10)	5 (18)	3 (11)	2 (6)	
**CRS**					
**Any grade**	81 (89)	19 (68)	28 (100)	34 (97)	
**Grade 1-2**	79 (87)	19 (68)	27 (96)	33 (94)	
**Grade 3+**	2 (2)	0 (0)	1 (4)	1 (3)	
**ICANS**					
**Any grade**	54 (59)	12 (43)	17 (61)	25 (71)	
**Grade 1-2**	26 (29)	6 (21)	10 (36)	10 (29)	
**Grade 3+**	28 (31)	6 (21)	7 (25)	15 (43)	

^Kruskal-Wallis rank-sum test, ‡Fisher's exact test

medRxiv preprint doi: https://doi.org/10.64898/2026.03.16.26348550; this version posted March 18, 2026. The copyright holder has placed this preprint (which was not certified by peer review) in the Public Domain. It is no longer restricted by copyright. Anyone can legally share, reuse, remix, or adapt this material for any purpose without crediting the original authors.

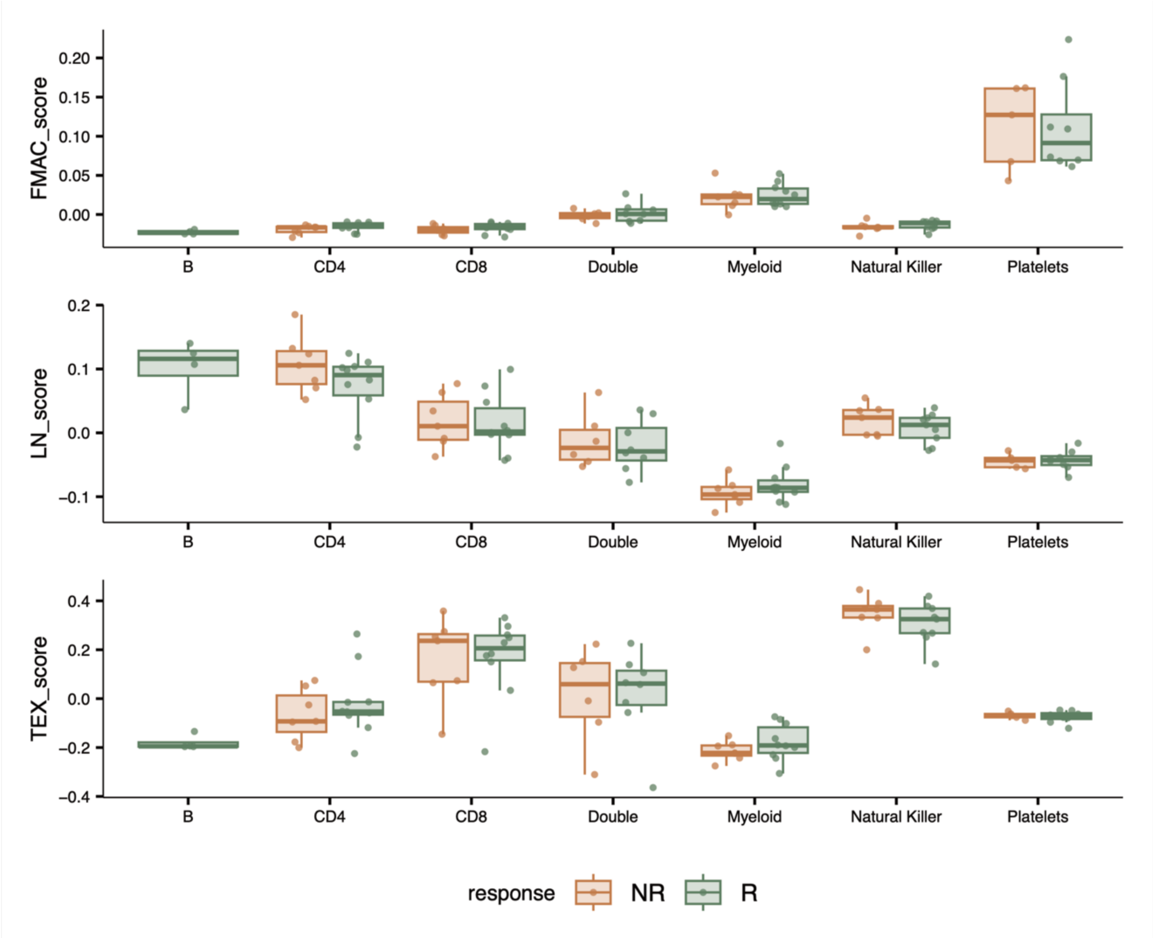

Figure S1. TME archetype module scores across PBMC cell types. FMAC (top), LN (middle), and TEX (bottom) archetype module scores computed from pseudobulk scRNA-seq profiles of major PBMC cell types, comparing responders (R, green) and non-responders (NR, orange). Sample-cell type pairings with less than 10 cells were removed from the analysis.

medRxiv preprint doi: https://doi.org/10.64898/2026.03.16.26348550; this version posted March 18, 2026. The copyright holder has placed this preprint (which was not certified by peer review) in the Public Domain. It is no longer restricted by copyright. Anyone can legally share, reuse, remix, or adapt this material for any purpose without crediting the original authors.

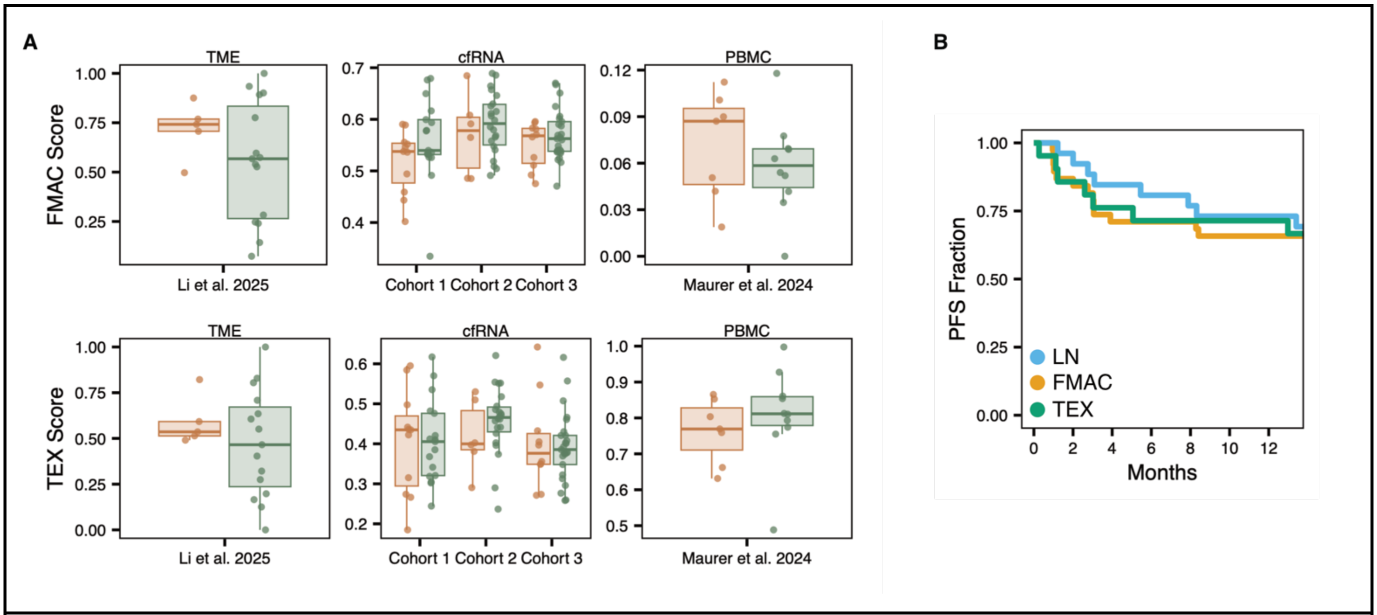

Figure S2. FMAC and TEX archetype scores and LymphoMap classification. A) FMAC (top) and TEX (bottom) archetype module scores in responders (green) and non-responders (orange) across TME biopsies (Li et al. 2025), plasma cfRNA (Cohorts 1-3), and PBMCs (Maurer et al. 2024). **B)** Progression-free survival curves for patients classified by the LymphoMap algorithm (Li et al. 2025) applied to cfRNA data. Patients were assigned to LN (blue), FMAC (orange), or TEX (green) groups.

medRxiv preprint doi: https://doi.org/10.64898/2026.03.16.26348550; this version posted March 18, 2026. The copyright holder has placed this preprint (which was not certified by peer review) in the Public Domain. It is no longer restricted by copyright. Anyone can legally share, reuse, remix, or adapt this material for any purpose without crediting the original authors.

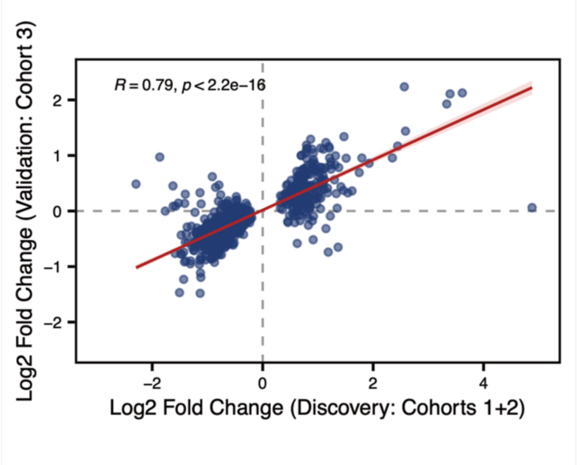

Figure S3. Correlation of $\log_2$ fold changes for transcripts differentially abundant between responders and non-responders in the discovery cohort (Cohorts 1 and 2) compared with the independent validation cohort (Cohort 3). Red line indicates linear regression fit with 95% confidence interval (Pearson R = 0.79, p < 2.2e-16).

medRxiv preprint doi: https://doi.org/10.64898/2026.03.16.26348550; this version posted March 18, 2026. The copyright holder has placed this preprint (which was not certified by peer review) in the Public Domain. It is no longer restricted by copyright. Anyone can legally share, reuse, remix, or adapt this material for any purpose without crediting the original authors.

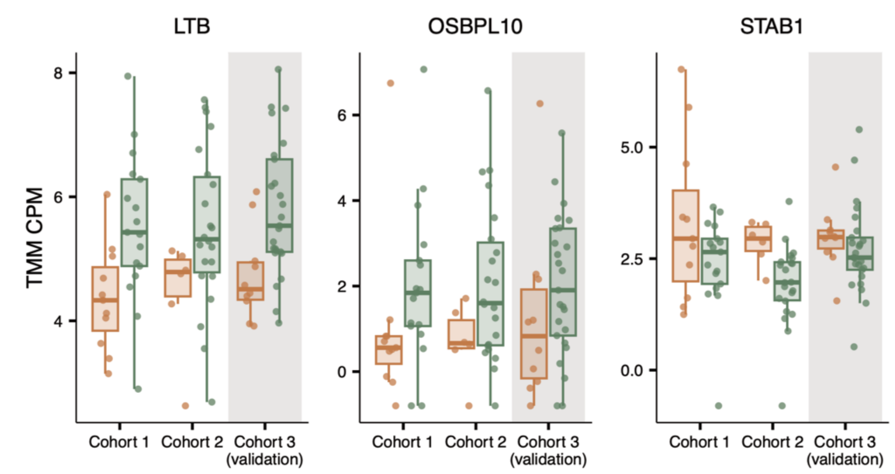

Figure S4. Differentially abundant marker transcripts in cfRNA. Normalized cfRNA abundance (log2 TMM CPM) of *LTB*, *OSBPL10*, and *STAB1* across all three cohorts. Grey shading indicates the validation cohort (Cohort 3).
